# Is dispatcher-assisted cardiopulmonary resuscitation affected by a bystander’s emotional stress state in out-of-hospital cardiac arrest?

**DOI:** 10.1186/s13049-023-01117-6

**Published:** 2023-11-17

**Authors:** Rebecca Hvidt Tuffley, Fredrik Folke, Annette Kjær Ersbøll, Stig Nikolaj Fasmer Blomberg, Gitte Linderoth

**Affiliations:** 1 Copenhagen Emergency Medical Services, University of Copenhagen, Telegrafvej 5, Ballerup, 2750 Denmark; 2 Dept. of Cardiology, Copenhagen University Hospital – Herlev and Gentofte, Borgmester Ib Juuls Vej 1, Herlev, 2730 Denmark; 3 National Institute of Public Health, University of Southern Denmark, Studiestraede 6, Copenhagen, 1455 Denmark; 4 Dept. of Anesthesiology, Copenhagen University Hospital – Bispebjerg and Frederiksberg, Bispebjerg Bakke 23, Copenhagen, 2400 Denmark; 5 Dept. of Clinical Medicine, University of Copenhagen, Blegdamsvej 3B, Copenhagen, 2200 Denmark

**Keywords:** Dispatcher-assisted CPR, Cardiopulmonary resuscitation, Out-of-hospital resuscitation, OHCA, Resuscitation, Chest compression

## Abstract

**Aim:**

The study aimed to investigate whether a bystander’s emotional stress state affects dispatcher-assisted cardiopulmonary resuscitation (DA-CPR) in out-of-hospital cardiac arrest (OHCA). The primary outcome was initiation of chest compressions (Yes/No). Secondarily we analysed time until chest compressions were initiated and assessed how dispatchers instructed CPR.

**Method:**

The study was a retrospective, observational study of OHCA emergency calls from the Capital Region of Denmark. Recorded calls were evaluated by five observers using a pre-defined code catalogue regarding the variables wished investigated.

**Results:**

Included were 655 OHCA emergency calls, of which 211 callers were defined as emotionally stressed. When cardiac arrest was recognized, chest compressions were initiated in, respectively, 76.8% of cases with an emotionally stressed caller and 73.9% in cases with a not emotionally stressed caller (2.18 (0.80–7.64)). Cases with an emotionally stressed caller had a longer time until chest compressions were initiated compared to cases with a not emotionally stressed caller, however non-significant (164 s. vs. 146 s.; P = 0.145). The dispatchers were significantly more likely to be encouraging and motivating, and to instruct on speed and depth of chest compressions in cases with an emotionally stressed caller compared to cases with a not emotionally stressed caller (1.64 (1.07–2.56); 1.78 (1.13–2.88)). Barriers to CPR were significantly more often reported in cases with an emotionally stressed caller compared to cases with a not emotionally stressed caller (1.83 (1.32–2.56)).

**Conclusion:**

There was no significant difference in initiation of chest compressions or in time until initiation of chest compressions in the two groups. However, the dispatchers were overall more encouraging and motivating, and likely to instruct on speed and depth of chest compressions when the caller was emotionally stressed. Furthermore, barriers to CPR were more often reported in cases with an emotionally stressed caller compared to cases with a not emotionally stressed caller.

**Trial registration:**

We applied for ethical approval from The Danish National Committee on Health Research Ethics, but formal approval was waived. We received permission for storage of data and to use these for research of OHCAs in the Capital Region of Denmark by Danish Data Protection Agency (P-2021-670) and Danish Health Authorities (R-2,005,114). The study is registered at ClinicalTrials (NTC05113706).

**Supplementary Information:**

The online version contains supplementary material available at 10.1186/s13049-023-01117-6.

## Introduction

Out-of-hospital cardiac arrest (OHCA) is a global public health challenge. The annual incidence of OHCA in Europe is 67–170 per 100,000 inhabitants [1]. On average, only 8% of patients survive OHCA [1]. OHCA outcome depends on the chain of survival, as a survival rate of 20–40% can be reached when OHCA is witnessed and early defibrillation is initiated [2].

Early and correct recognition of OHCA by the emergency medical dispatcher is pivotal for initiation of bystander cardiopulmonary resuscitation (CPR). Dispatcher-assisted CPR (DA-CPR) has been shown to increase bystander CPR and have a positive effect on OHCA outcomes [3–9]. Recognizing OHCA can be difficult and even experienced medical dispatchers only recognize OHCA in approximately 75% of calls [8].

However, the interaction between the medical dispatcher and caller can be affected by the caller’s state of emotional stress. The caller’s state of emotional stress can be caused by numerous reasons such as the severity of the situation, relation to the patient and the demeanor of the medical dispatcher [10]. This has been suggested to make the medical dispatcher’s interrogation unsuccessful and hence delay recognition of OHCA [11]. However, previous studies have shown that only 8.4% of OHCA emergency callers had a high emotional stress score, whereas 90% of the callers were in fact cooperative during the emergency call [11, 12].

We aimed to investigate whether a bystander’s emotional stress state affects DA-CPR in OHCA. The primary outcome was initiation of chest compressions (Yes/No). Secondarily we analysed time until chest compressions were initiated and assessed how the dispatchers instructed CPR.

## Method

### Setting

The Emergency Medical Dispatch Center in Copenhagen, Denmark, serves 1.8 million inhabitants [13]. The emergency calls are initially handled by a call center, which identifies the caller’s location and forwards the call to a medical dispatcher. The medical dispatchers are trained registered nurses and paramedics. In case of OHCA, the medical dispatcher should guide the bystander to perform CPR until the arrival of an ambulance and to localize the nearest AED. The DA-CPR instructions given vary with the caller’s CPR experience, from compression-only CPR to conventional CPR. Furthermore, nearby volunteer citizen responders are activated to help with CPR and deliver an AED before arrival of emergency medical services (EMS) [14].

### Study design

The study was a retrospective cohort study of all emergency calls with recognized OHCA from the Capital Region of Denmark during the time period September 1st, 2018 until November 1st, 2019. The Strengthening the Reporting of Observational studies in Epidemiology guidelines (STROBE) were consulted during the conduction of the study [15]. The data used were obtained from the study by Blomberg, S. N. et al. [16]. The study identified validated OHCA cases through the Danish Cardiac Arrest Registry [17] and the related audio emergency call recordings were obtained from the EMS voice-log database. Only OHCA’s recognized by a medical dispatcher were included. All EMS-witnessed OHCA’s and calls where the caller was not physically by the patient’s side were excluded.

### Data collection

The rating of the calls was performed by five trained evaluators outside of the research group who had no specific medical background and no conflict of interests. The five evaluators registered data from the emergency calls using a pre-specified code catalogue. The code catalogue was a standardized and objective instruction for the observers, which specifically listed all variables wished addressed, and criteria for each category [See Additional File 1].

Prior to assessing the calls, the group was presented to real emergency calls for rating consensus and instructions. The interrater reliability was evaluated for bystander’s emotional stress and symptoms, in 16 randomly selected emergency calls of confirmed OHCA. A Kappa score of overall agreement of 0.72 was reached, which provides substantial agreement between the observers [18]. According to the code catalogue, the evaluation of the bystander’s emotional stress state was based on a simplified emotional stress symptom-based score. The score was based on the emotional content and cooperation score (ECCS) [10], which is used to evaluate the emotion and cooperation of the caller. The ECCS is classified in five levels, ranging from normal conversational speech to uncontrollable, hysterical bystanders [10]. However, during validation of the five level score, we found the interobserver variability to be too high. The score was then modified to an emotional stress symptom-based score, due to the intention of decreasing the interobserver variability (a higher kappa score) and enhancing a practical usefulness of the score. The callers were registered binary as either “*Emotionally stressed*” or “*Not emotionally stressed*”. Emotional stress was defined as: “*If the caller seemed emotionally affected by the situation. If the caller spoke with shallow patting, affected tone of voice other than just rapid speech or if the caller expressed worries concerning the victim and was afraid of the outcome*”. Furthermore, symptoms of emotional stress were registered (*“Caller was crying”, “Caller was screaming”*, and *“Caller was not paying attention because of emotional stress”*).

Furthermore, variables concerning the dispatcher’s CPR instructions were also registered using the pre-specified code catalogue. The dispatchers’ CPR instructions were evaluated based on whether the dispatchers were assertive or passive when giving CPR instructions, whether they were encouraging and motivating, if they instructed on speed and depth of chest compressions, and whether they addressed an AED. The assessment of whether the dispacther was assertive or passive was based on the following “*Indicate in the text box whether the dispatcher was “Assertive” or “Passive” in his/her effort to give CPR instructions. Dispatchers who ask callers “Are you willing to do CPR?” or “Do you want to try CPR?”, for example, are Passive. Dispatchers who tell callers, “We need to start CPR” or “I need you to start CPR” are Active.”.* The assessment of whether the dispatcher was encouraging was based on the following “*Coded as Yes, if encouraging or motivating techniques are in use, e.g. «keep on going», «you’re doing a great job»,”the ambulance is on its way”. Encouraging or motivating techniques must be ongoing throughout the call, it is not enough to just say «keep on going» once*”. All criterions for each of the abovementioned variables are further elaborated in the code catalogue (Additional file 1).

### Outcome

We aimed to investigate whether a bystander’s emotional stress state affects DA-CPR in OHCA. The primary outcome was initiation of chest compressions (Yes/No). Secondarily we analysed time until chest compressions were initiated and assessed how the dispatcher instructed CPR.

### Ethics

We applied for ethical approval from The Danish National Committee on Health Research Ethics, but formal approval was waived. We received permission for storage of data and to use these for research of OHCAs in the Capital Region of Denmark by Danish Data Protection Agency (P-2021-670) and Danish Health Authorities (R-2,005,114). The study is registered at ClinicalTrials (NTC05113706).

### Analysis

Descriptive analysis of baseline characteristics of emergency calls and dispatchers was performed by frequency distributions (N %) stratified by emotional stress. Kappa statistics was used to assess inter-rater variability and agreement between the reviewers.

The association between a caller’s emotional stress state and chest compression initiated (Yes/No) was analysed using logistic regression. It was not considered relevant to adjust for variables such as “*Caller’s sex”*, “*Was caller alone at time of call*” and “*Caller’s relation to the patient*” since the variables are not directly related to the outcome wished investigated. Results are presented as odds ratio (OR) with a corresponding 95% confidence interval (95% CI).

A Wilcoxon rank sum test was used on non-normally distributed continuous variables to analyse differences in time until chest compressions were initiated according to the caller’s emotional stress state. Results were reported as median time, percentages and p-value. A p-value < 0.05 was considered significant.

Logistic regression was used to evaluate the dispatcher’s CPR instructions according to caller’s emotional stress state. Results were reported as odds ratios (OR) with a 95% CI. Data were analysed using R-studio (V 1.4.1717 for Mac, © 2009–2021 R-Studio, PBC).

## Results

**Fig. 1:**
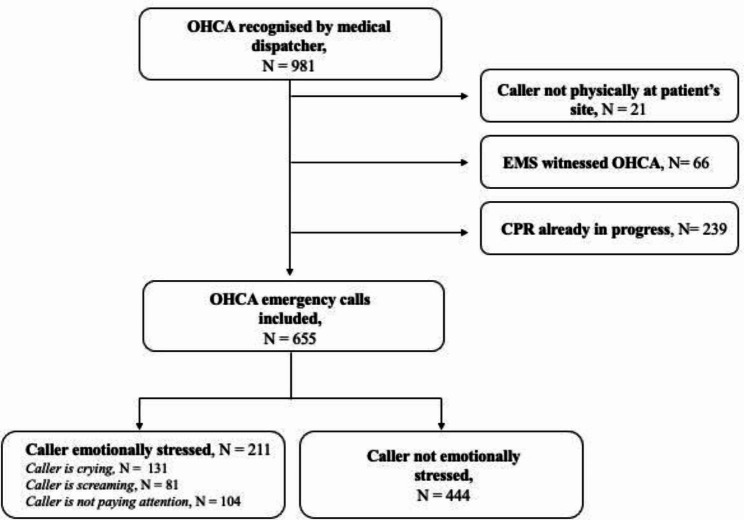
Flowchart. In total, 326 calls were excluded due to the caller was not physically by the patient’s site, EMS witnessed OHCA or CPR already in progress

In total, 981 OHCA emergency calls were obtained, of which 87 calls were excluded due to EMS witnessed cardiac arrest or caller not being at the patient’s site. Another 239 calls were excluded due to CPR already being in progress when the emergency call was initiated (Fig. 1).

Out of all 655 calls, 211 (32.2%) callers were emotionally stressed, whereas 62.1% were registered ad “*Caller is crying*”, 38.4% were registered as “*Caller is screaming*”, and 49.3% were registered as “*Caller is not paying attention*” as seen in Table 1. Some callers were registered with multiple symptoms.

Most callers were female in both cases with an emotionally stressed caller (76.3%) and cases with a not emotionally stressed caller (60.1%). Compared to a not emotionally stressed caller, the emotionally stressed callers were: more often relatives to the patient (85.8% vs. 40.5%), less often healthcare professionals (2.3% vs. 33.1%), and more often alone at the time of the call (59.2% vs. 38.3%), respectively (Table 1).

The median age of patients in calls with an emotionally stressed caller was lower than in calls where the caller was registered as not emotionally stressed (68.5 years vs. 78.0 years). There was a higher incidence of return of spontaneous circulation (ROSC) in the calls with an emotionally stressed caller (35.1% vs. 25.0%). Additional characteristics of the calls are shown in Table 1.

Furthermore, we analyzed potential variables that might impact the callers emotional stress level using a multivariate logistic regression adjusting for “*Caller’s sex*”, “*Was caller alone at time of call*” and “*Caller’s relation to the patient*”. We found a significant impact on the caller’s emotional stress states when the caller was female or non-healthcare professional (Relative and other) (0.34 (0.21–0.55); 48.04 (17.09-201.28); 11.52 (3.70–50.80).

**Table 1:** Characteristics of out-of-hospital cardiac arrest (OHCA) emergency calls according to the caller’s emotional stress state. N/A represents the cases where it was not possible to register a value

Emergency call characteristics	Caller emotionally stressed (N = 211 (32.2%))	Caller not emotionally stressed (N = 444 (67.8%))
**Caller emotionally stressed and:**		
***is crying***	131 (62.1%)	
***is screaming***	81 (38.4%)	
***is not paying attention***	104 (49.3%)	
**Caller’s sex**		
***Female***	161 (76.3%)	267 (60.1%)
***Male***	50 (23.7%)	176 (39.6%)
***N/A***	0 (0%)	1 (0.3%)
**Caller’s relation to patient**		
***Healthcare professional***	5 (2.3%)	147 (33.1%)
***Relative***	181 (85.8%)	180 (40.5%)
***Other***	25 (11.9%)	117 (26.4%)
**Was caller alone at time of call**		
***Yes***	125 (59.2%)	170 (38.3%)
***No***	86 (40.8%)	274 (61.7%)
***N/A***	0 (0%)	0 (0%)
**ROSC**		
***Yes***	74 (35.1%)	111 (25.0%)
***No***	117 (55.4%)	286 (64.4%)
***N/A***	20 (9.5%)	47 (10.6%)
**Patient’s age**		
***Median (25–75% quantile)***	68.5 (57–81)	78.0 (67–86)

### Chest compressions initiated and time until initiation

Chest compressions were initiated in 490 of all included emergency calls, of which 162 (76.8%) were initiated in calls with an emotionally stressed caller and 328 (73.9%) were initiated in calls with a not emotionally stressed caller (2.18 (0.80–7.64)) (Table 2).

Time until chest compressions were initiated was longer in cases with an emotionally stressed caller compared to cases with a not emotionally stressed caller, however non-significant (164 s. vs. 146 s.; P = 0.145) (Table 2).

**Table 2:** Chest compressions initiated and time until chest compressions were initiated. Results of logistic regression as odds ratio (OR) with a 95% confidence interval (CI) is shown. Time until chest compressions initiated were analyzed using Wilcoxon rank sum test presented with a P-value

Chest compressions initiated	Yes (N (%))	CI (95% CI)	
***Caller emotionally stressed***	162 (76.8%)	2.18 (0.80–7.64)	
***Caller not emotionally stressed***	328 (73.9%)	Ref.	
**Median times until OHCA recognized, CPR instructions initiated and chest compressions initiated**			
**Median time until (seconds)**:	**Caller emotionally stressed (sec.)**	**Caller not emotionally stressed (sec.)**	**P-Value**
***CA recognized***	73	74	P = 0.641
***CPR instructions initiated***	112	119	P = 0.453
***Chest compressions initiated***	164	146	P = 0.145

### Assessment of the dispatcher’s cpr instructions

The dispatchers were significantly more likely to be encouraging and motivating, and to instruct on speed and depth of chest compressions in cases with an emotionally stressed caller compared to cases with a not emotionally stressed caller (1.64 (1.07–2.56); 1.78 (1.13–2.88)) (Table 3).

**Table 3:** The performance of dispatcher-assisted cardiopulmonary resuscitation (DA-CPR) according to caller’s emotional stress state. Results of logistic regression as odds ratio (OR) with a 95% confidence interval (CI) is shown

	N (%)	OR (95% CI)
**Dispatcher is being assertive**		
***Caller emotionally stressed***	158 (74.9%)	1.55 (0.97–2.56)
***Caller not emotionally stressed***	297 (66.9%)	Ref.
**Dispatcher is being encouraging and motivating**		
***Caller emotionally stressed***	131 (62.1%)	1.64 (1.07–2.56)
***Caller not emotionally stressed***	239 (53.8%)	Ref.
**Dispatcher is instructing on speed and depth of compressions**		
***Caller emotionally stressed***	138 (65.4%)	1.78 (1.13–2.88)
***Caller not emotionally stressed***	253 (57.0%)	Ref.
**Dispatcher addresses an AED**		
***Caller emotionally stressed***	61 (28.9%)	0.80 (0.56–1.14)
***Caller not emotionally stressed***	150 (33.8%)	Ref.

### Barriers to resuscitation

Barriers to CPR was more often reported in cases with an emotionally stressed caller compared to cases with a not emotionally stressed caller (58.3% vs. 43.2%; 1.83 (1.32–2.56)). Both groups reported “*Could not move patient*” as the most frequent barrier (33.2% and 20.3%; 1.95 (1.35–2.82)) (Table 4). Further barriers are listed in Table 4.

**Table 4:** Barriers to dispatcher assisted cardiopulmonary resuscitation (DA-CPR) according to the caller’s emotional stress state. Results of logistic regression as odds ratio (OR) with a 95% confidence interval (CI) and P-value is shown

	Caller emotionally stressed, 211 (N (%))	Caller not emotionally stressed, 444 (N (%))	OR (95% CI)
**Barrier to CPR**	123 (58.3%)	192 (43.2%)	1.83 (1.32–2.56)
**Could not move patient**	70 (33.2%)	90 (20.3%)	1.95 (1.35–2.82)
**Language barrier**	3 (1.4%)	6 (1.3%)	1.05 (0.22–4.03)
**Caller left phone**	18 (8.5%)	23 (5.2%)	1.71 (0.89–3.22)
**Caller hangs up phone**	5 (2.4%)	9 (2.0%)	1.17 (0.36–3.44)
**Caller refused**	21 (10.0%)	26 (5.9%)	1.77 (0.96–3.23)
**Patient’s status changes**	11 (5.2%)	34 (7.6%)	0.66 (0.31–1.29)
**Do not resuscitate order**	4 (1.9%)	5 (1.1%)	1.69 (0.41–6.47)
**Patient obviously dead**	10 (4.7%)	19 (4.3%)	1.11 (0.49–2.38)
**Other**	8 (3.8%)	13 (2.9%)	1.30 (0.51–3.15)
**N/A**	10 (4.7%)	24 (5.4%)	0.87 (0.39–1.80)

N/A represents the cases where the evaluator was not able to register if barriers to CPR were present

## Discussion

We aimed to investigate whether a bystander’s emotional stress state affects DA-CPR in OHCA. The primary outcome was initiation of chest compressions (Yes/No). Secondarily we analysed time until chest compressions were initiated and assessed how the dispatcher instructed CPR.

We found no significant difference in initiation of chest compressions according to the caller’s emotional stress state. There was a longer time until initiation of chest compressions in cases with an emotionally stressed caller compared to cases with a not emotionally stressed caller, however, non-significant. We found that the dispatchers were significantly more likely to be encouraging and motivating, and to instruct on speed and depth of chest compressions during resuscitation attempt, in cases with an emotionally stressed caller compared to cases with a not emotionally stressed caller.

There was a higher incidence of ROSC in the cases where the caller was emotionally stressed. Even though there was a non-significantly longer time until chest compressions were initiated, the outcome of OHCA was better in the cases with an emotionally stressed caller. This could imply that being an emotionally stressed bystander, somewhat might be favorable in OHCA. However, one could imagine that reaching a certain level of emotional stress might not be beneficial. The Yerkes-Dodson law states that moderate arousal can actually enhance performance, although high levels of arousal can decrease performance [19]. However, the patients were generally younger in the cases where the caller was emotionally stressed, which would, expectedly, improve the probability of OHCA outcome.

Although only 32.2% of the callers were emotionally stressed, many of them actually presented symptoms such as crying, screaming, or not paying attention, implying that some callers were in fact severely emotionally stressed. Previous studies have primarily used an ECCS score of the original five groups, but without specified symptoms [11, 22]. To investigate whether the symptoms used in this study have an impact on OHCA variables, further research in the area is needed.

Many of the not emotionally stressed callers consisted of healthcare professionals, who would be expected to be less stressed. Alfsen et al. analyzed emergency calls regarding OHCA and found that when the caller is a healthcare professional, the roles were reversed, and the responsibility was given to the caller. This resulted in a longer time until recognition of OHCA due to the CA algorithm being abandoned [20]. The emotionally stressed callers were primarily relatives to the patient. This could result in a higher emotional stress state. Studies have found that relatives less often provide CPR, even though they received instructions from the dispatcher [21]. We found that the caller cases with an emotionally stressed caller, were more often alone, which could also be an important factor in the higher emotional stress state.

The longer time until chest compressions were initiated could be explained by the emotional stress causing confusion or delayed actions taken. However, a similar study evaluating emergency call recordings regarding OHCA by Chien et al. found a shorter median time until recognition of OHCA and first chest compression in caller’s registered as ECCS 4–5 compared to ECCS 1–3 [11]. Our study only evaluated the caller’s emotional stress state in two categories, and more elaborate classification might explore further differences.

Dispatchers were significantly more likely to be encouraging and motivating, to instruct on speed and depth of chest compressions during resuscitation attempt, in cases with an emotionally stressed caller compared to cases with a not emotionally stressed caller. This might be due to the medical dispatcher sensing that the caller was emotionally stressed and therefore tried to provide more precise and encouraging instructions. 27% of callers were characterized as “*Emotionally stressed*”, meaning the vast majority of the callers were calm (ECCS 1–2) (73%). Various studies on the impact of stress coping strategy during simulated CPR and real OHCA audio recordings also found 90% of the callers to be cooperative (ECCS 1–3) [22, 23].

Furthermore, we found that emotionally stressed callers more often reported barriers to DA-CPR. The most frequent barrier was “*Could not move the patient*”. Wah Ho et al., also found this to be the most prevalent barrier [9]. Others have found the most prevalent barrier to be “*Caller refused*” [11]. An emotionally stressed caller could assess the situation under the influence of their emotional stress and report a barrier that might be manageable to eliminate. Furthermore, Wah Ho et al. suggested that barriers to DA-CPR are related to a longer time until chest compressions initiated and to be related to lower proportions of chest compressions eventually being performed [9]. Lewis et al. found that the factors delaying recognition of OHCA were related to the dispatcher asking unnecessary questions, and the caller’s emotional stress state [24]. A study by Calle et al. on the effect of training medical dispatchers showed a 20% increase in collecting relevant information before vs. after training [25].

### Limitations

The study only analyzed the dispatcher assisted resuscitation and not the recognition of cardiac arrest, which might also be affected by a bystander’s emotional stress. Unfortunately, many of the calls did not have registered times until chest compressions were initiated. This might be due to difficulty determining this through audio recordings. Previous studies have shown a thorough insight to OHCA cases using live video streaming from bystander’s smartphones, which might give a more precise timestamp until any of the abovementioned events [26].

Additionally, even though an overall kappa score of 0.72 was reached, suggesting substantial agreement amongst observers, rating the callers is a case of individual assessment. The assessment depends on the individual observers’ evaluation, which provides a degree of unintended subjectivity.

Being assertive, motivating and encouraging is in this study presented as being a positive factor when performing DA-CPR. Dispatchers in Denmark follow a criteria-based protocol termed the Danish Index for Emergency Care, helping the dispatcher assess the severity of the call [27]. However, this protocol does not instruct the dispatcher to be encouraging or assertive, etc. In a study of qualitative interviews of 11 medical dispatchers, Møller et al. suggested that non-technical skills training of medical dispatchers should be implemented along with the medical expertise skills [28].

The study cannot determine the quality of DA-CPR, since we only registered whether the dispatchers were being assertive or passive, encouraging and motivating, instructed on speed and depth of chest compressions, and addressed an AED. The amount of guidance and quality of this would be difficult using this binary method (Yes/No) when registering data. A qualitative study design might address this in a more precise manner.

## Conclusion

There was no significant difference in chest compressions initiated and time until chest compressions were initiated, when comparing cases with respectively an emotionally stressed caller and a not emotionally stressed caller.

However, we found that dispatchers were significantly more likely to be encouraging and motivating, and to instruct on speed and depth of chest compressions during resuscitation attempt, in cases with an emotionally stressed caller compared to cases with a not emotionally stressed caller.

### Electronic supplementary material

Below is the link to the electronic supplementary material.

Supplementary Material 1

## Data Availability

The data that support the findings of this study are not openly available due to reasons of sensitivity and are available from the corresponding author upon reasonable request.
