# Supplementary material for: Is dispatcher-assisted cardiopulmonary resuscitation affected by a bystander’s emotional stress state in out-of-hospital cardiac arrest?
Source: Scand J Trauma Resusc Emerg Med. 2023 Nov 17;31:82. doi: 10.1186/s13049-023-01117-6 (PMC10656878; doi:10.1186/s13049-023-01117-6)
Supplement: Supplementary file 1 — Supplementary Material 1 [file 13049_2023_1117_MOESM1_ESM.docx]

**APPENDIX 1: Code Catalogue**

**1. INITIAL**

**Definition:**

- This is the initials of the employee that annotated the call.

**Instructions for Coding:**

- Spell out the full name of the employee or use a standardized abbreviation. Please choose one or the other.
- Upper case letters are preferred.
- Do not use periods, commas, or semicolons.

**Examples:**

NB

NIKOLAJ BLOMBERG

**2. EVA-NUMBER**

**Definition**:

- This is the number sequentially assigned to the call by the Dispatch Agency’s CAD. The EVA-number of the call are retrieved from list of calls to annotate.

**Description:**

- The EVA-number serves as a call’s unique identifier within a Dispatch Agency. It is usually sufficient for linking dispatch and pre-hospital records.
- This field is not nullable. A unique value must be provided to create a unique record ID within
- the database.

**Instructions for Coding:**

- Enter the EVA Number assigned to the call.

**Examples:**

**EVA**

RGH-4905477

**3. TIME OF DISPATCHER ADRESSING CALLER (MM:SS)**

**Definition:**

- This is the time after police transfer of call and adress, when the dispatcher introduces herself to the caller.

**Description:**

- When recording starts, the police are usually still talking to the caller. This is proceeded by a very brief conference, where the police confirms the adress. Upon this the dispathcer will adress the caller.

**Instructions for Coding:**

- Enter in minutes (“MM”) and seconds (“SS”) the elapsed time from the start of the call to the moment the dispatcher adress the caller.

**4. CPR ALREADY IN PROGRESS?**

**Definition:**

- CPR is “already in progress” when callers indicate that they or other lay or trained rescuers on scene have started CPR before the dispatcher starts instructions for CPR.

**Description:**

- Calls where CPR is already in progress should be excluded when calculating the proportion of cases where dispatchers recognize the need for CPR, start CPR instructions, and achieve the first bystander compression. They should also be excluded when calculating the median or average time to these events from the start of the call.

**Instructions for Coding:**

- If CPR is known to start before a call-taker or dispatcher start instructions for CPR, mark the circle next to “Yes” under “CPR already in progress?”
- If CPR does not start before a call-taker or dispatcher starts instructions for CPR, mark the circle next to "No.”
- If it is not known whether CPR started before a call-taker or dispatcher started instructions for CPR, mark the circle next to “Unknown.”

**Example:**

**Is CPR in progress?**

O Yes

O No

O Unknown

**5. CONSCIOUSNESS ADRESSED**

**Definition:**

- Consciousness is adressed by dispatcher or caller at any given time during the call

**Description:**

- A patient’s level of consciousness is a key indicator of whether he or she is in cardiac arrest. It can be difficult to get a clear answer on whether the patient is conscious. Callers often give contrary answers to this question at different times in the call. Type-appropriate CPR instructions should be given when a patient is deemed not conscious and not breathing normally.

**Instructions for Coding:**

- If signs of consciousness is addressed by either dispatcher or caller (”awake,” ”conscious,” ”contact,” ”reactions,” etc , mark the circle next to “Yes” under “ Consciousness addressed”
- If the dispatcheror caller does not adress consciousness, mark the circle next to "No.”
- If the patient is patient obviously dead or dispatcher initiate CPR without adressing consciousness, mark the circle next to “N/A.”

Remember, indications are NOT sufficient, e.g. "completely gone" or "he collapsed"

**6. PATIENT IS CONSCIOUS?**

**Definition:**

- A patient is considered conscious if the caller reports the patient is conscious and/or responsive to the caller. A patient is considered not conscious if the caller reports the patient is not

conscious and/or is not responsive to the caller.

**Description:**

- A patient’s level of consciousness is a key indicator of whether he or she is in cardiac arrest. It can be difficult to get a clear answer on whether the patient is conscious. Callers often give contrary answers to this question at different times in the call. Type-appropriate CPR instructions should be given when a patient is deemed not conscious and not breathing normally.

**Instructions for Coding:**

- Mark the circle next to the appropriate answer (“Yes,” No,” or “Unknown”) under “Conscious?”.

**Examples:**

- The caller says her husband is “passed out and not responding.” Mark the “No” circle, coding the patient as not conscious.
- The caller does not commit in answering whether the patient is conscious, saying “yes” at one point, “no” at another and “I can’t tell” at another. The dispatcher asks if she can speak with the patient. The caller says, “No, there’s no way he can talk to you.” If the caller reports that the patient can’t speak, it indicates the patient is most likely not conscious. Mark the “No” circle, coding the patient as not conscious.
- A caller says the patient is in a seizure. The seizure then stops, and the caller reports that the patient “is snoring like he’s in a deep sleep and he won’t wake up.” A patient who “won’t wake up” should be classified as not conscious. Mark the circle next to “No”, coding the patient as not conscious.
- The caller reports the patient wouldn’t wake up a minute ago, but now appears to be “getting better.” The dispatcher tells the caller to shake the patient’s shoulders to see if the patient responds. The caller says he moaned and pushed her arms away. A patient who makes purposeful movement (pushing the caller’s arms away) is demonstrating conscious intent and should be coded as conscious. Mark the circle next to “Yes”.

**7. BREATHING ADRESSED**

**Definition:**

- Breathing is adressed by dispatcher or caller at any given time during the call

**Description:**

- .

**Instructions for Coding:**

- If breathing is addressed by either dispatcher or caller, mark the circle next to “Yes” under “ Breathing addressed”
- If the dispatcheror caller does not adress breathing, mark the circle next to "No.”
- If the patient is patient obviously dead or dispatcher initiate CPR without adressing breathing, mark the circle next to “N/A.”

**8. PATIENT IS BREATHING NORMALLY?**

**Definition:**

- A patient is considered to be breathing normally if the caller reports the patient is breathing normally. A patient is considered to be breathing not normally if the caller reports the patient is (A) not breathing or (B) the caller reports abnormal breathing and/or (C) the Quality Assurance (QA) rater hears abnormal breathing and/or identifies it through the caller’s description of the patient’s breathing. Abnormal breathing is defined as breathing with a rate and/or character different from the victim’s normal breathing at rest.

**Description:**

- A patient’s breathing status is a key indicator of whether he or she is in cardiac arrest. It can be difficult to get a clear answer on whether the patient is breathing normally. Callers often give contrary answers to this question at different times in the call. Agonal breathing is very common in cardiac arrest. Callers often use specific words or phrases to describe this kind of breathing. These descriptions include, but are not limited to, “gasping,” “gasping for air,” “gurgling,” “gargling,” “snoring,” “snorting,” “humming,” “moaning,” “groaning,” “breathing every once in a while” and “shallow breathing.” Type-appropriate CPR instructions should be given when a patient is deemed not breathing normally and not conscious.

**Instructions for Coding:**

- Mark the circle next to the appropriate answer (“Yes,” No,” or “Unknown”) under “Breathing Normally?” on the “Dispatch: Patient” section of the QA form. In cases where callers describe agonal breathing or where the quality assurance rater hears agonal breathing, patients should be coded as not breathing normally.

**Examples:**

- The caller says her husband is drunk and that he keeps “gurgling and gasping for air.” The descriptors “gurgling and gasping for air” indicate agonal breathing. Even if the caller suspects it’s just because her husband is drunk, mark the “No” circle, coding the patient as not breathing normally.
- The caller says his wife “seems to be breathing okay,” but the quality assurance rater hears a soft snoring sound in the background. The dispatcher does not hear it or hears it but does not identify it as abnormal breathing. Mark the “No” circle, coding the patient as not breathing normally.

**9. DOES THE DISPATCHER ADRESS NORMAL/ABNORMAL BREATHING**

**Definition:**

- When breathing are adressed, the dispatcher.

**Instructions for Coding:**

- If the dispatcher addresses normal/abnormal breathing, mark the circle next to ”Yes”. This includes following up if caller initially mentions the patient's breathing
- If the dispatcher does not address normal/abnormal breathing, mark the circle next to ”No”. This includes if the dispatcher only asks "Is the patient breathing?"

**10. DID DISPATCH RECOGNIZE NEED FOR CPR?**

**Definition:**

- A dispatcher or call-taker recognizes the need for CPR when he or she indicates that CPR should be performed in the course of the call.

**Description:**

- The dispatcher recognizes the need for CPR when he or she says any of the following in connection with a response to the victim’s condition: “CPR,” “chest compressions,” “compressions,” “continuous chest compressions,” “CCR,” “rescue breaths,” “rescue breathing,” “ventilations,” or “rescue ventilations.” In some cases, the dispatcher might not say any of these but indicates recognition by starting CPR instructions. In such cases, the time to dispatch recognition of the need for CPR and the time to start of CPR instructions are the same.

**Instructions for Coding:**

- If the dispatcher indicates that he or she recognizes the need for CPR, mark the circle next to “Yes” under “Did dispatch recognize the need for CPR?”
- If the dispatcher does not indicate that he or she recognizes the need for CPR, mark the circle next to "No.”
- If it is not known whether the dispatcher indicated recognition of the need for CPR, mark the circle next to “Unknown.”

**Example:**

**Did dispatch recognize the need for CPR?**

O Yes

O No

O Unknown

**11. TIME OF RECOGNITION (of need for CPR)**

**Definition:**

- The time dispatch recognizes the need for CPR is the time elapsed from the start of the call to the moment when the dispatcher or call-taker indicates that he or she realizes CPR should be performed.

**Description:**

- Dispatcher and call-taker recognition of the need for CPR is the first of three key time intervals in the provision of pre-arrival CPR instructions.
- Dispatchers and call-takers indicate their recognition when they say any of the following in connection with a response to the patient’s condition: “Cardiopulmonary Resuscitation,” “CPR,” “chest compressions,” “compressions,” “continuous chest compressions,” “Hands-Only CPR,” “CCR,” “rescue breaths,” “rescue breathing,” “ventilations,” or “rescue ventilations.” In some cases, the dispatcher might not say any of these but indicates recognition by starting CPR instructions. In such cases, the time to dispatch recognition of the need for CPR and the time to start of CPR instructions are the same.
- If the dispatcher or call-taker indicates his or her recognition, but subsequently instructs the caller or rescuer either to “lift the patient’s chin and tilt his or her head back” and/or “to look, listen and feel for breathing,” the time to dispatch recognition of the need for CPR should be defined as the moment the dispatcher or call-taker indicates his or her recognition AFTER instructing the caller or rescuer to perform this formal breathing assessment.

**Instructions for Coding:**

- Enter in minutes (“MM”) and seconds (“SS”) the elapsed time from the start of the call to the moment of dispatch recognition of the need for CPR.

**Examples:**

- The dispatcher says, “We need to start CPR right away.” Enter the time elapsed to the moment when the dispatcher says “CPR.”
- The dispatcher says, “We need to start CPR” at 1 minute and 27 seconds into the call. She then instructs the caller to lift the patient’s chin, tilt his head back and to look, listen and feel for breathing. The caller performs this procedure. It takes 25 seconds, and at 1:52 the dispatcher says, “OK, let’s start compressions.” Enter 1:52 as the time to dispatch recognition of the need for CPR.
- The patient is on the floor and the caller describes him as “not conscious” and “not breathing normally.” A second later, at 55 seconds, the dispatcher then says, “kneel by his side and put the palm of one hand in the center of his chest. Put your other hand on top of that hand.” The dispatcher has not said “CPR” or anything synonymous. He has launched directly into the start of CPR instructions. In this case, the time elapsed to dispatch recognition is the same as the time elapsed to the start of dispatch instructions: 55 seconds.

**12. BLS COMPETENCE ADRESSED**

**Definition:**

- When the dispatcher adress the callers ability to perform CPR.

**Description:**

- The dispatcher adress BLS competence when he or she ask the caller if they are trained in CPR. Adressing willingsness to perform CPR is not fulfilling the criteria .

**Instructions for Coding:**

- If the dispatcher ask the caller if he or she has been trained in CPR, mark the circle next to “Yes”
- If the dispatcher does adress the callers competence, mark the circle next to "No.”

**13. CPR INSTRUCTIONS STARTED?**

**Definition:**

- CPR instructions are directions dispatchers and call-takers provide to guide callers through the process of performing CPR, whether compression-only or conventional CPR (CPR with rescue breathing). Instructions are considered “started” if they are simply started, even if they are not finished.

**Description:**

- Instructions to get a patient to a hard, flat surface should not be considered the start of CPR instructions. In many protocols, instructions start when a call-taker or dispatcher tells the rescuer to “kneel by the patient’s side”. The moment when CPR instructions are considered started, however, may vary from one dispatch center to another according to language used in local protocols.

**Instructions for Coding:**

- If CPR instructions are started, mark the circle next to “Yes” under “CPR instructions started?”
- If CPR instructions are not started, mark the circle next to "No.”
- If it is not known whether CPR instructions were started, mark the circle next to “Unknown.”

**Examples:**

- A caller is ready to start CPR. The dispatcher begins instructions, saying, “Kneel by the patient’s side,” but the caller stops him abruptly, saying the patient is “waking up and is conscious now.” The dispatcher does not continue the CPR instructions he started. Code as “Yes.” Although CPR instructions were stopped just after they were started in this example, they were still started.

**CPR instructions started**

O Yes

O No

O Unknown

**14. TIME OF INSTRUCTIONS STARTED (MM:SS) (DISPATCHER BEGAN INSTRUCTIONS)**

**Definition:**

- This is the time elapsed from the start of the call (or in the case of a Transfer Call, the time elapsed from the moment the dispatcher or call-taker first addresses the caller) to the moment when the dispatcher or call-taker starts CPR instructions.

**Description:**

- The time at which a dispatcher or call-taker starts CPR instructions is the second key time interval in the provision of pre-arrival instructions. This method for assigning this time will vary from dispatch center to dispatch center, depending on the wording of protocols. Instructions to get a patient to a hard, flat surface should not be considered the start of CPR instructions. In many protocols, instructions begin when a call-taker or dispatcher tells the rescuer to “kneel by the patient’s side.”

**Instructions for Coding:**

- Enter in minutes (“MM”) and seconds (“SS”) the elapsed time from the start of the call to the moment the dispatcher or call-taker starts CPR instructions.

**Examples:**

- The caller reports that she is ready to start CPR. The dispatcher says, “kneel by his side and put the palm of one hand in the center of his chest ,” at 2 minutes and 12 seconds. Enter 2:12 as the time at which the dispatcher began instructions for CPR.

**15. BARRIERS TO CPR?**

**Definitions:**

- Barriers to CPR are defined as obstacles that prevent the start of dispatch-directed, bystander chest compressions. They include:
  - Hang up phone: This is when the caller disconnects from the dispatcher or call-taker processing the call.
  - Language barrier: This when the caller and dispatcher do not speak the same language and therefore cannot communicate effectively.
  - Caller left phone: This is when the caller leaves the phone for purposes other than rendering aid to the patient after speaking with the dispatcher or call-taker.
  - Caller not with patient: This is when the caller is speaking from a location that prohibits the caller’s physical assessment of patient.
  - Overly distraught: This is when a caller’s highly-distressed emotional state delays or prevents him or her from taking CPR instructions and/or performing CPR.
  - Caller refused: This is when a dispatcher or call-taker suggests or instructs CPR and a caller refuses for reasons other than a physical inability to perform CPR.
  - Couldn’t move patient: This is when a caller reports his or her inability to move the patient from an unsuitable location for CPR (e.g., toilet or bed).
  - Patient status change: This is when a patient initially thought to be in cardiac arrest presents indication that he or she is not in cardiac arrest.
  - Obviously dead: Caller conveys that patient is deceased. In this case, the caller provides sufficient evidence to the dispatcher in support of that conclusion (e.g. rigor mortis, mottled skin, decomposition, foul odor).
  - Other: Any barrier apart from those defined above that prevents the start of CPR instructions and/or bystander chest compressions

**Description:**

- Barriers to CPR are important to track because the recurrence of given barriers can point the way to protocol changes addressing high-frequency obstacles. For example, a common barrier is that rescuers can’t move a patient from a bed to a suitable location where compressions could be effective. Knowing this, managers and medical directors can experiment with protocol language and procedures to help rescuers solve this problem.
- Multiple barriers can delay or prevent the start of CPR in any one call.

**Instructions for Coding:**

- Check the box next to the appropriate item under “Barriers to CPR” according to the definitions above.

**Examples:**

- The caller, a native Spanish speaker, speaks and understands English poorly. The dispatcher knows little Spanish, but is able to get the caller to do CPR after several minutes of trying to clarify his instructions. Code as a delay to start of CPR resulting from “Language barrier”
- The dispatcher tries to calm a hysterical caller, but the caller screams and then leaves the phone. The caller is heard screaming in the background until EMTs arrive. Code as “Overly distraught” and “Caller left phone”
- A dispatcher tells the caller that she needs to start CPR and that he will help her. The caller refuses, however, saying she has hurt her back and that there is no way she can get the patient from the bed to the floor. Code as “Other” (and what that “other” barrier was: physical inability). The caller has refused to take CPR instructions but for reasons owing to a physical inability to perform (her bad back). Code as “Couldn’t move patient”
- The patient appears to be unconscious in the back yard, but the caller is on a landline phone on the second floor of the house. The caller is thus not able to physically assess the patient’s status. Code as “Caller not with patient”
- The caller reports that the patient is not conscious and not breathing normally. The dispatcher starts instructions for CPR, but the patient opens his eyes and begins to mumble and deliberately starts rubbing his head. The dispatcher recognizes the patient is conscious and discontinues CPR instructions. Code of “Patient status change”
- The caller indicates that the patient is not conscious and not breathing normally. The dispatcher starts instructions for CPR, but the caller subsequently says the patient is “blue, cold and stiff as a board.” The dispatcher discontinues CPR instructions. Code as “Obviously dead”

**16. IS THE DISPATCHER ASSERTIVE OR PASSIVE WHEN GIVING CPR INSTRUCTIONS?**

**Definition:**

**Description:**

Evaluate how assertive the dispatcher or call-taker was in getting instructions started once he or she identified the need for CPR. It is equally important to point out those things a dispatcher or call-taker does well when handling a suspected cardiac arrest call.

**Instructions for Coding:**

- Indicate in the text box whether the dispatcher was “Assertive” or “Passive” in his/her effort to give CPR instructions. Dispatchers who *ask* callers “Are you willing to do CPR?” or “Do you want to try CPR?”, for example, are Passive.
- Dispatchers who *tell* callers, “We need to start CPR” or “I need you to start CPR” are Active.

**Examples:**

- The dispatcher was Passive. He asked if the caller wanted to do CPR instead of telling him, “We need to start CPR.”
- The dispatcher was Assertive. He told the caller “We need to start CPR.”

**17. IS THE DISPATCHER INSTRUCTING ON SPEED AND DEPTH OF COMPRESSIONS**?

**Description:**

Evaluate the quality of instructions in regards to speed and depths when dispatcher is giving instructions on CPR.

• Yes, when dispatcher comments on speed and depth

• No

• N/A (No CPR)

**Instructions for Coding:**

•Coded as «Yes» if dispatcher is checking quality of CPR. E.g. «please count out loud with me», «are you pushing deep enough?» «push a bit faster/slower»

- Yes, if encouraging or motivating techniques are in use
- No, if no encouraging or motivating techniques are in use
- Unknown
- N/A

**18. IS THE DISPATCHER USING ENCOURAGING AND MOTIVATING TECHNIQUES WHEN INSTRUCTING?**

**Description:**

Evaluate the instructions in regards to motivation and encouragement when dispatcher is giving instructions on CPR.

**Instructions for Coding:**

- Coded as Yes, if encouraging or motivating techniques are in use, e.g. «keep on going», «you’re doing a great job», ”the ambulance is on its way”
- Encouraging or motivating techniques must be ongoing throughout the call, it is not enough to just say «keep on going» once

**19. CHEST COMPRESSIONS STARTED?**

**Definition:**

- Chest compressions are considered “started” if a rescuer does *any* chest compressions, even if the rescuer stops just after starting.

**Description:**

- Determining whether chest compressions are started can be difficult in a minority of cases. Rescuers don’t always count out their compressions, and sometimes their voices or the compressions themselves are inaudible.

**Instructions for Coding:**

- If chest compressions were started, mark the circle next to “Yes” under “Chest Compressions Started?”
- If chest compressions were not started, mark the circle next to "No.”
- If it is not known whether chest compressions were started, mark the circle next to “Unknown.”

**Examples:**

**Chest compressions were started**

O Yes

O No

O Unknown

**20. TIME TO FIRST COMPRESSION**

**Definition:**

- This is the time elapsed from the start of the call (or in the case of a Transfer Call, the time elapsed from the moment the dispatcher or call-taker first addresses the caller) to the moment when the caller or rescuer delivers the first chest compression.

**Description:**

- The time to first compression is the third of three key time intervals in the provision of prearrival CPR instructions. The time is noted when the first compression is audible or the caller/rescuer indicates he or she has started compressions (i.e. by counting with dispatcher).

**Instructions for Coding:**

- Enter in minutes (“MM”) and seconds (“SS”) the elapsed time from the start of the call to the moment the caller or rescuer delivers the first chest compression. There are often calls in which the time to first compression must be carefully inferred or entered as “Unknown.”

**Examples:**

- The dispatcher finishes instructions for starting compressions, and the caller clearly counts out the first compression at 3 minutes and 23 seconds into the call. Enter the time elapsed to first compression as 3:23.
- The dispatcher finishes instructions for CPR at 2 minutes and 50 seconds into the call and tells the caller to count the compressions out loud. The caller doesn’t count, however, and, eight seconds later, at 2:58, the dispatcher asks, “Are you doing the compressions?” The caller says, “Yes.” The dispatcher then reminds the caller to count out loud, and the caller begins: “1, 2, 3 …” In this scenario, it becomes clear that the caller is doing CPR at 2:58 seconds (the caller says, “Yes” when asked if he’s doing compressions.) The dispatcher told him to count out loud at 2:50. Since 8 seconds later the caller said he had been doing compressions, it can be reasonably inferred that the first compression occurred somewhere between 2:51 and 2:55. In the absence of more perfect information, enter the elapsed time as 2:53, the midpoint between 2:51 and 2:55.
- The dispatcher finishes instructions for CPR and tells the caller to count out loud at 1:46. The caller doesn’t count, but the first of a string of audible compressions occurs at 1:49. Enter the time elapsed to first compression as 1:49.

**21. WAS AN AED ADRESSED?**

**Description:**

Thsi is when either the dispatcher or caller mentions an AED.

**Instructions for Coding:**

- Yes - If an AED or similar wording referring to an AED is mentioned by either caller or dispatcher
- No - If an AED or similar is not mentioned
- N/A (when OHCA is not recognized)

**22. WAS AN AED CONNECTED TO THE PATIENT?**

- Yes - If the caller actively says that he/she or someone else has connected the AED to the patient, or it is clearly heard that the AED is on and gives instructions to the bystander.
- No - If none of the above occurred.
- N/A

**23. DID THE AED DELIVER A SHOCKTO THE PATIENT?**

- Yes - If the caller actively says that the AED has delivered shock/shocks, or it is clearly heard that the AED is giving instructions to deliver a shock.
- No - If none of the above occurred.
- N/A - If you answered no or N/A to number 20 or are in doubt.

**24. WAS THE CARDIAC ARREST WITNESSED BY ANOTHER PERSON**

- yes - If the caller actively says that he/she or someone else saw the patient collapse, or if the caller or someone else heard the patient collapse and were by their side within seconds.
- No - If none of the above occurred.
- N/A

**25. WAS THIS A CARDIAC ARREST BEFORE ARRIVAL OF EMS?**

**Definition:**

- A suspected cardiac arrest is confirmed or not confirmed by Emergency Medical Technicians when they assess the patient’s status upon arrival.

**Description:**

- Call-takers and dispatchers may misidentify medical events as cardiac arrests based on a caller’s description. Only calls linked to EMS-confirmed cardiac arrests should be evaluated for qualityimprovement purposes.

**Instructions for Coding:**

- If the patient is unconciuos, not breathing etc. during the call, mark the circle next to “Yes”
- If it is clearly heard that the patient is talking, concious, breathing etc. during the call, mark the circle next to “No.”

**Example:**

**If a Cardiac Arrest**

O Yes

O No

O Unknown

**26. CALL CONTINUED UNTIL EMS ARRIVAL**

- Yes - If it is addressed or clearly heard, that the ambulance personnel is by the patient
- No - If the call is terminated before the ambulance personnel arrives at the side of the patient, or if the medical dispatcher sees that the ambulance personnel has pushed “arrived” and ends the call, or the sirens is heard in the recording and call is ended.
- N/A

**27. CALLER SEX**

- Female
- Male
- N/A

**28. WAS THE CALLER ALONE AT THE TIME OF CALL?**

- Yye
- No
- N/A

**29. CALLER RELATION TO PATIENT**

**Definition:**

- The callers relation to the person, whether it is a caller that knows the patient, a caretaker or a completely stranger passing by

**Description:**

- Callers fall into a variety of categories. Caller can Only calls linked to EMS-confirmed cardiac arrests should be evaluated for qualityimprovement purposes.

**Instructions for Coding:**

- If the patient is related to the patient (Mother, father, grandparents, uncle, aunt, cousin, friends of the family etc.), mark the circle next to “Yes”
- If caller has healthcare education/background regardless of level (MD, nurse, paramedic, emergency medical technician, Social - and Health Service Helper/assistant, social worker at institutions, any of the above in training) mark the circle next to “Healthcare”. If healthcare professional is related to the patient mark both.
- Other - If caller does not fit in to one of the above, mark the circle next to “Other”

**30. CALLERS ACCESS TO ASSESMENT OF PATIENT**

- Is by the patient's side - If direct visual contact to the patient, can see and/or touch the patient without leaving the phone.
- Can access the patient - If caller can potentially see and touch the patient but has to leave the phone to do so, no visual contact, communication through third party.
- Cannot access the patient - If access to the patient is impossible in the current situation.

**31&32. CALLER EMOTIONALLY DISTRESSED**

**Definition:**

- Determining emotional distress. “Caller” is the first person the dispatcher talks to. When caller is emotional distressed multiple codes can be registered.

**Instructions for Coding:**

Caller emotional distressed?: **Yes or** No.

“Yes” (if caller seems emotional affected by the situation). Determining if caller is emotional affected can be difficult. If caller speaks with shallow patting, affected tone of voice other than just rapid speech or if caller express worries concerning the victim and is afraid of the outcome “caller emotional distressed” should be coded.

**“**No” (if caller has normal conversational speech or speaks faster than expected for a normal conversation, but otherwise do not sound emotional distressed).


How does the caller show emotional distress?

- Caller is crying: This is when you can hear that caller is crying or snuffling.
- Caller is shouting/screaming: This is when caller is shouting/screaming in the phone because of emotional distress. (This code should not be used if caller is shouting for other reasons than emotional distress, i.e. because of loud background noise or if caller is shouting to either the victim or to other bystanders to check the victims’ level of consciousness).
- Caller did not pay attention to questions and/or instructions raised from dispatcher: This is when caller at any giving time during the first minute does not pay attention to what the dispatcher says. Examples: If the dispatcher has to repeat questions/instructions or if caller does not respond to questions raised by the dispatcher (This code should only be used if caller is emotional distressed and not be used if callers lack of attention is due to noise or if caller just asks the dispatcher to repeat the question/instructions).

Mark the box next to the appropriate item under “Are the bystanders emotional distressed**”**” according to the definitions above.

**Examples:**

| Ex. Caller says: “*I am so afraid of losing him*” or “*This is what I feared”* or “*This cannot be true*”.  Caller has shallow panting | Code as “Caller emotional distressed” |
| --- | --- |
| Caller is yelling: “My Husband is all blue. Send an ambulance”. Caller has shallow panting. No crying is heard. | Code as “Caller emotional distressed” and ““Caller is shouting/screaming”. |
| Caller shouts:” *You have to send an ambulance right away. No, No, This is not happening. No No.”* Caller is crying. | Code as “Caller is shouting/screaming”, “Caller emotional distressed” and ”Caller is crying”. |
| Dispatcher:” *What is your address*”. Caller” *he just collapsed*”. Dispatcher: “*I need to know your address”.* After 30 sec. caller has shallow panting. | Code as “Caller did not pay attention to questions/instructions” and “Caller emotional distressed”. |
| Caller is crying | Code as “Caller is crying” and caller “emotional distressed” |

**33. OTHER COMMENTS?**

**Definition:**

Other Comments refer to any thoughts the evaluator may have with respect to research and process improvement ideas, or comments in relation to the soundquality, abrupt ending or such.

**Instructions for Coding:**

- Enter free text comments.
